# Anti-*Anopheles darlingi *saliva antibodies as marker of *Plasmodium vivax *infection and clinical immunity in the Brazilian Amazon

**DOI:** 10.1186/1475-2875-8-121

**Published:** 2009-06-05

**Authors:** Bruno Bezerril Andrade, Bruno Coelho Rocha, Antonio Reis-Filho, Luís Marcelo Aranha Camargo, Wanderli Pedro Tadei, Luciano Andrade Moreira, Aldina Barral, Manoel Barral-Netto

**Affiliations:** 1 Centro de Pesquisas Gonçalo Moniz FIOCRUZ – Bahia, Brazil; 2 Faculdade de Medicina da Bahia, Universidade Federal da Bahia, Brazil; 3 Centro de Pesquisas René Rachou, FIOCRUZ – Belo Horizonte, Minas Gerais, Brazil; 4 Unidade Avançada de Pesquisa, Instituto de Ciências Biológicas V, Universidade de São Paulo, São Paulo, Brazil; 5 Faculdade de Medicina, Faculdade São Lucas, Rondônia, Brazil; 6 Laboratório de Malária e Dengue, Instituto Nacional de Pesquisa da Amazônia – Manaus, Amazonas, Brazil; 7 Instituto Nacional de Ciência e Tecnologia de Investigação em Imunologia (iii), Salvador, Bahia, Brazil

## Abstract

**Background:**

Despite governmental and private efforts on providing malaria control, this disease continues to be a major health threat. Thus, innovative strategies are needed to reduce disease burden. The malaria vectors, through the injection of saliva into the host skin, play important role on disease transmission and may influence malaria morbidity. This study describes the humoral immune response against *Anopheles (An.) darlingi *saliva in volunteers from the Brazilian Amazon and addresses the association between levels of specific antibodies and clinical presentation of *Plasmodium (P.) vivax *infection.

**Methods:**

Adult volunteers from communities in the Rondônia State, Brazil, were screened in order to assess the presence of *P. vivax *infection by light microscopy and nested PCR. Non-infected volunteers and individuals with symptomatic or symptomless infection were randomly selected and plasma collected. *An. darlingi *salivary gland sonicates (SGS) were prepared and used to measure anti-saliva antibody levels. Plasma interleukin (IL)-10 and interferon (IFN)-γ levels were also estimated and correlated to anti-SGS levels.

**Results:**

Individuals infected with *P. vivax *presented higher levels of anti-SGS than non-infected individuals and antibody levels could discriminate infection. Furthermore, anti-saliva antibody measurement was also useful to distinguish asymptomatic infection from non-infection, with a high likelihood ratio. Interestingly, individuals with asymptomatic parasitaemia presented higher titers of anti-SGS and lower IFN-γ/IL-10 ratio than symptomatic ones. In *P. vivax*-infected asymptomatic individuals, the IFN-γ/IL-10 ratio was inversely correlated to anti-SGS titers, although not for while in symptomatic volunteers.

**Conclusion:**

The estimation of anti-*An. darlingi *antibody levels can indicate the probable *P. vivax *infection status and also could serve as a marker of disease severity in this region of Brazilian Amazon.

## Background

Malaria continues to be one of the most serious public health problems worldwide, exacting a huge impact on human wellbeing, mainly in tropical and subtropical countries. A better understanding of the interactions between the host, the vector and the parasite could be valuable to indicate future strategies. In endemic regions, residents are frequently bitten by both uninfected and infected mosquitoes. There is also a progressive acquisition of immunity, leading to a decreased number of malaria clinical attacks related to increasing age and time residing in the endemic area [1,2]. Within the Brazilian Amazon, and mainly in riverine communities, the prevalence of asymptomatic malaria infection seems to be four to five times greater than the symptomatic infection [3-5]. Malaria clinical immunity has already been described in both *Plasmodium (P.) falciparum *[6] and *Plasmodium (P.) vivax *[7] infections and it seems to be related to higher titers of anti-*Plasmodium *antibodies [8]. On the other hand, anti-parasite response might not be the unique determinant of the occurrence of symptomless malaria, as asymptomatic patients maintain parasitaemia at low levels in addition to controlling the clinical symptoms [9]. Such asymptomatic carriers have developed just enough immunity to protect them from malarial illness but not from malarial infection. Regardless these facts, the specific mechanisms that underlie the occurrence of clinical immunity against the *Plasmodium *are not well understood.

In this scenario, the anopheline vector could play significant role in malaria clinical severity. Mosquito bites can induce immediate, delayed, and systemic hypersensitivity reactions in hosts [10]. Moreover, pre-exposure to the vector saliva may create an inhospitable environment for the establishment of the parasites transmitted by these insects. Mice repeatedly exposed to bites from uninfected *Anopheles (An.) stephensi *increase a pro-inflammatory T helper 1 biased response that limits *P. yoelii *infection [11]. In humans it has been shown that *An. gambiae *saliva is immunogenic for travelers transiently exposed to bites in African endemic areas [12], with the development of specific IgG and IgM antibodies. Specific anti-*An. gambiae *saliva IgG antibodies were also detected in young children from a seasonal malaria transmission region in Senegal, and antibody levels were higher in patients who developed clinical malaria episodes, suggesting that the estimation of humoral response to *Anopheles *salivary antigens can serve as potential marker for the risk of malaria [13]. Moreover, anti-*An. dirus *salivary protein antibodies occur predominantly in patients with acute *P. falciparum *or *P. vivax *malaria, whereas people from non-malarious areas do not carry such antibodies [14]. Little is known about anti-saliva humoral responses in other endemic areas, such as Latin America. In addition, the host response against the most widespread malaria vector in America, *An. darlingi*, is poorly explored. The objective of the present work was to measure the anti-saliva IgG responses against *An. darlingi *mosquitoes in the Brazilian Amazon and to evaluate the association of antibody levels with different clinical presentations of *P. vivax *infections.

## Methods

### Study localities

A cross-sectional study investigating determinant factors for asymptomatic *P. vivax *malaria was performed during 2007 (June to August) in Buritis (10°12'43" S; 63°49'44" W), a recent urbanized municipality, and Demarcação (8°10'04.12" S; 62°46'52.33" W), a riverine community of the Rondônia State, in the south-western part of Brazilian Amazon. In general, Rondônia has a flat topography, with an average elevation of 300 m above sea level. The climate is tropical, with a long rainy season from January till May. It is argued that the environmental changes caused by deforestation have favored the main malaria vector in Brazil *An. darlingi *[15]. Within the regions studied here, the malaria transmission is unstable, with increased number of cases being detected annually between April to September, and the risk of infection is moderate to high [16], with an Annual Parasite Incidence of 77.5 per 1,000 inhabitants in 2005 [17]. In the Brazilian Amazon, *P. vivax *accounts for the majority of malaria cases, while *P. falciparum *infection prevalence is 23.7% [17]. In addition, infection with *P. malariae *achieves 10% in Rondônia [18].

### Volunteers

Active and passive malaria case detections were performed in the two communities studied. A small laboratory with necessary facilities was built inside the main centers for malaria diagnosis in Buritis and Demarcação. These diagnostic centers are linked to the Brazilian National Foundation of Health (FUNASA), responsible for malaria control in the Brazilian Amazon. Active case detection was made by visiting residences in regions pointed by the local health authorities as major areas of disease transmission. The individuals were examined and interviewed by a trained physician, and blood samples were collected for serological experiments. The malaria diagnosis was performed using two methods. First, patients were screened by thick smear examination using field microscopy and the parasitaemia (parasites/μL) was calculated in positive cases. Further, nested PCR was performed in all whole blood samples to confirm the diagnosis (as described below). Two individuals presenting *P. malariae *infection and 16 persons infected with *P. falciparum *were identified and excluded from the study. Hence, all the volunteers selected were negative for *P. falciparum *and/or *P. malariae *infection by both microscopic examination and nested PCR. Other exclusion criteria were chronic alcoholism, severe chronic degenerative disease as well as HIV, HBV and HCV infections. A total of 204 volunteers were used in the study. All the positive cases were followed up for 30 days for the evaluation of malaria symptoms. Individuals who were positive for *P. vivax *infection and remained without fever (axilary temperature >37.8°C) and/or chills, sweats, strong headaches, myalgia, nausea, vomiting, jaundice, asthenia, and arthralgia for 30 days were considered asymptomatic, while in the presence of any listed symptom they were classified as symptomatic. The volunteers were stratified in three different groups according to the *P. vivax *malaria diagnosis and the clinical spectrum of the disease. Thus, 80 people were non-infected, 50 had asymptomatic infection and 74 were symptomatic. The baseline characteristics of the volunteers are listed in the Table 1. Three volunteers from asymptomatic infection group presented negative light microscopy exam, but *P. vivax *DNA was amplified by nested PCR (Table 1). This study was a part of the project approved by the Ethical Committee of the São Lucas University, Rondônia, Brazil, for the human subject protocol and is in compliance with the Helsinki Declaration. All participants gave written informed consent before entering the study.

**Table 1:** Baseline characteristics of the volunteers.

		*Plasmodium vivax *current infection		P value
		
Variable	Non-infected	Asymptomatic	Symptomatic	
	(n = 80)	(n = 50)	(n = 74)	
Age – years*	30 (23–44.5)	44.5 (34.5–51)	27.5 (21–37)	0.0341†
Malaria episodes referred*	13.5 (11–18)	17.5 (13–21)	7 (1–13)	0.0283†
Time residing in the area – years				0.0185‡
<2	25 (31.3%)	8 (16%)	31 (41.9%)	
3–10	12 (15%)	12 (24%)	16 (21.6%)	
>10	43 (53.7%)	30 (60%)	27 (36.5%)	
Parasitaemia – parasites/μL				< 0.0001‡
ND§	80 (100%)	3 (6%) §	0	
100–<500	0	44 (88%)	34 (45.9%)	
500–<5,000	0	3 (6%)	5 (6.8%)	
5,000–<50,000	0	0	30 (40.5%)	
>50,000	0	0	5 (6.8%)	
IgG anti-SGS – O.D.*	0.06 (0.04–0.09)	0.13 (0.08–0.26)	0.095 (0.07–0.14)	< 0.0001†
Plasma IL-10 – pg/mL*	12.6 (7.4–19.2)	64.5 (7.3–86.0)	23.4 (9.5–58.4)	NS†
Plasma IFN-γ – pg/mL*	14.2 (0–32.0)	44.0 (10.5–101.0)	75.5 (38.8–243.5)	NS†

* Values plotted represent media and range

† Ordinal variables were compared between groups Kruskal-Wallis test with Dunn's multiple comparison test.

‡ Categorized variables were compared using chi-squared test. P values obtained in each test are plotted.

§ND: Six patients, out of 50 were negative for malaria infection by light microscopy, but were positive for *P. vivax *infection by nested PCR.

NS: Non significant.

### Molecular malaria diagnosis

The molecular diagnosis of malaria infection was performed using the nested PCR technique, based on the Snounou protocols, with minimal alterations [19,20]. The target was the 18S rRNA gene, and genus- and species-specific primers were used in the assay. Briefly, 300 μL of whole blood collected on EDTA was prepared for DNA extraction through the phenol-chloroform method followed by precipitation with sodium acetate and ethanol. The first PCR rDNA amplification was performed with *Plasmodium *genus-specific primers named PLU5 and PLU6. Positive samples yielded a 1,200-bp fragment, which served as template for the nested reaction. The nested PCR amplification was performed with species-specific primers for 30 cycles at annealing temperatures of 58°C for *P. falciparum *(Fal1 and Fal2 primers), and 65°C for *P. vivax *(Viv1 and Viv2 primers) or *P. malariae *(Mal1 and Mal2 primers). The fragments obtained for *P. vivax *were of 120 bp, whereas for *P. falciparum *and *P. malariae *were 205 bp and 144 bp, respectively. The oligonucleotide sequences of each primer used are listed in Table 2. The products were visualized in 2% agarose gel stained with ethidium bromide. To control for cross-contamination, one uninfected blood sample was included for every twelve samples processed. Fifteen percent of positive PCR samples were re-tested to confirm the amplification of plasmodial DNA. All the tests were performed and confirmed at the Centro de Pesquisas Gonçalo Moniz (FIOCRUZ-BA).

**Table 2:** Primers used in Nested PCR reactions.

Primer	Oligonucleotide Sequence 5'-3'	Base pairs
PLU5	CCTGTTGTTGCCTTAAACTTC	1,200
PLU6	TTAAAATTGTTGCAGTTAAAA	
Fal1	TTAAACTGGTTTGGGAAAACCAAATATATT	205
Fal2	ACACAATGAACTCAATCATGACTACCCGTC	
Viv1	CGCTTCTAGCTTAATCCACATAACTGATAC	120
Viv2	ACTTCCAAGCCGAAGCAAAGAAAGTCCTTA	
Mal1	ATAACATAGTTGTACGTTAAGAATAACCGC	144
Mal2	AAAATTCCCATGCATAAAAAATTATACAAA	

PLU: *Plasmodium sp*, Fal: *Plasmodium falciparum*, Viv: *Plasmodium vivax*, Mal: *Plasmodium malariae*.

### Salivary Gland Sonicate (SGS) preparation

Salivary glands from field captured adult female *An. darlingi *mosquitoes were dissected and transferred to 20 μL of 10 mM HEPES pH 7.0, 0.15 mM NaCl in 1.5-mL polypropylene vials, usually in groups of 20 gland pairs. Salivary glands were kept at -70°C until needed, when they were disrupted by sonication using a Branson Sonifier 450 homogenizer (Branson, Danbury, CT). The homogenates were centrifuged at 10,000 × g for 4 min and the supernatants were used for the experiments. Protein concentrations were measured by the bicinchonic acid method (BCA, Pierce, Rockford, Illinois, USA). As the salivary glands used in this study were obtained from field captured mosquitoes, *Plasmodium *contamination needed to be checked by nested PCR. Briefly, it was performed the DNA extraction of a sample from the same SGS pool used in the serological experiments using the Qiagen Generation Capture Card Kit (Cat. No. 159982; Qiagen, Santa Clara, California, USA). Further, the nested PCR was performed as described above, in duplicate samples. There was no amplification of DNA in both duplicates (data not shown).

### Anti-*An. darlingi *saliva serology

Volunteer's sera were collected and kept at -70°C. Serological tests of all samples were performed in a single experiment, with duplicate samples. ELISA was performed as described elsewhere [14]. Briefly, plates were coated with *An. darlingi *salivary homogenate (SGS) equivalent to 1.5 μg/mL in carbonate buffer overnight at 4°C, then washed with PBS/0.05% Tween and blocked with PBS/0.1% Tween plus 0.05% BSA. Sera were diluted 1:100 with PBS/0.05% Tween and incubated overnight at 4°C. After further washings, the wells were incubated with alkaline phosphatase-conjugated anti-human IgG (Sigma-Aldrich, St. Louis, MO) at a 1:5,000 dilution. Following another washing cycle, the color was developed with p-nitrophenylphosphate. The reactions were blocked with NaOH and read at 405 nm using Soft Max-Pro Software v5 (Molecular Devices Corporation, Sunnyvale, California, USA) ELISA reader. The optical density (OD) values plotted represent the means between each sample duplicate, adjusted for the values from the blank wells.

### Plasma cytokine measurement

Interleukin (IL)-10 and interferon (IFN)-γ plasma levels were measured using de Cytometric Bead Array – CBA^® ^(BD Biosciences Pharmingen, San Diego, California, USA) according to the manufacturer's protocol.

### Statistical analysis

Data were analyzed using the GraphPad Prism 5.00^® ^(GraphPad Software Inc.). For the ordinal variables (age, referred malaria episodes, IgG, IL-10 and IFN-γ serum levels), differences between groups were calculated using the non parametric Kruskal-Wallis test with Dunn's multiple comparison post test. Chi-square test was used to compare differences regarding categorized variables (Time residing in the area and parasitaemia). Mann-Whitney test was used to compare differences in IgG levels between non-infected individuals and those with symptomatic or asymptomatic *P. vivax *infection. This test was also used to estimate significance in IFN-γ/IL-10 ratios from volunteers with asymptomatic or symptomatic *P. vivax *infection. To evaluate the cut off value of IgG anti-SGS predicting malaria infection or asymptomatic infection, we performed Receiver-operator characteristic (ROC) curves, calculated the Area under curve (AUC), and then estimated the likelihood ratio for the discrimination between the conditions analyzed. Fine Lowess curves were plotted to evidence the trend of the data presented in correlation analyzes. Spearman test was used to verify the significance in the correlations between cytokine levels and anti-SGS levels. Differences were considered significant at P < 0.05.

## Results and discussion

**Figure 1:**
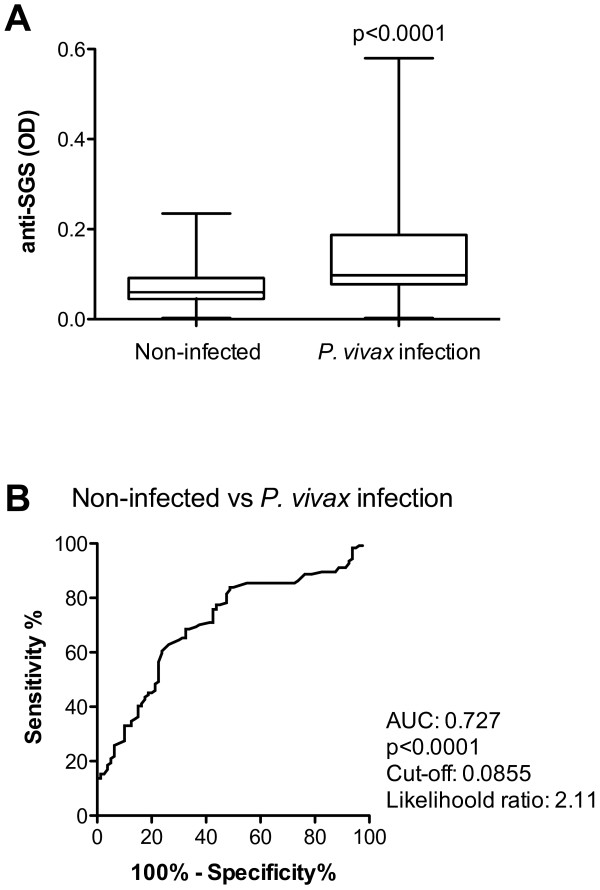
**Anti-saliva IgG serum levels according to malaria occurrence**. Serums were collected from non-infected individuals (n = 80), and from patients with *P. vivax *infection with or without symptoms (n = 124). An ELISA test was performed to assess the IgG anti-*An. darlingi *SGS. (A) Box plot graphs of IgG serum levels from non-infected individuals and from patients with *P. vivax *infection. Lines of the boxes represent 75^th ^percentile, median and 25^th ^percentile of the individual average OD values; whiskers represent the maximum and minimum values. Differences between groups were tested using Mann Whitney test. (B) ROC curve evaluating the threshold value of anti-SGS that separates non-infected individuals from *P. vivax *infection. Area under curve (AUC) calculated, together with the cut off value, which presents the higher likelihood ratio, and p values are plotted.

In an attempt to check if the measurement of anti-SGS antibody levels could be a suitable method to estimate natural exposure to *P. vivax*, anti-SGS values obtained from non-infected individuals were compared to those from either symptomatic or asymptomatic infected volunteers. As seen in Figure 1A, infected patients presented higher levels of specific antibodies against *An. darlingi *salivary antigens than non-infected individuals. The variability probably indicates individual differences in exposure to mosquito bites, even during the period of high malaria transmission, when these data were collected. Previous studies have also demonstrated a high variation in anti-*An. dirus *saliva antibody titers [14].

A ROC curve was built to assess the best anti-SGS OD value to discriminate *P. vivax *infection from the non-infected condition. A cut-off point of 0.0855 OD displayed a likelihood ratio to be infected of 2.11 indicating *P. vivax *infection (Figure 1B; AUC: 0.727; p < 0.0001). These data suggest that evaluation of anti-saliva antibodies could be a useful indicator to estimate exposure to *P. vivax *in this endemic area. High anti-SGS antibody levels were also proposed as putative biomarkers of exposure to bites of *An. stetephensi *or *An. gambiae *and also of risk of *P. falciparum *malaria [13]. In this study, besides suggesting exposure to bites, high anti-*An. darlingi *saliva antibody levels could also indicate exposure to *P. vivax*.

This work is the first to evaluate human immune response against salivary components of *An. darlingi*, the most widespread specie of *Anopheles *mosquitoes and the major malaria vector in the Americas [21]. In areas with unstable malaria transmission and moderate risk of infection, such as the Brazilian Amazon, adults, instead of children are largely affected by the disease. Hence, this study focused investigation on the adult population from a Brazilian endemic area.

**Figure 2:**
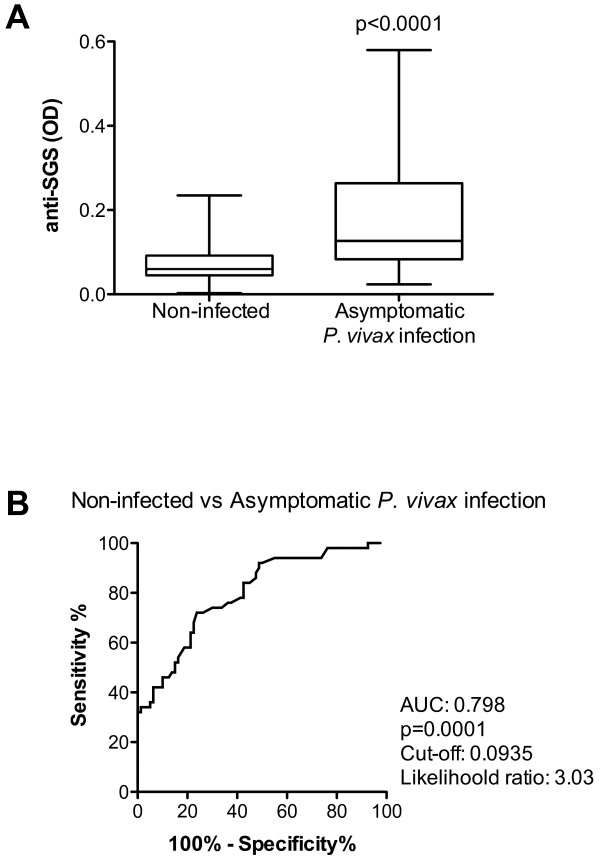
**Anti-saliva IgG serum levels from non-infected individuals and asymptomatic malaria patients**. Serums were collected from non-infected individuals (n = 80), and from patients with asymptomatic (n = 50) *P. vivax *infection. An ELISA test was performed to assess the IgG anti-*An. darlingi *SGS. (A) Box plot graphs of IgG serum levels from non-infected individuals and from patients with asymptomatic *P. vivax *infection. Lines of the boxes represent 75^th ^percentile, median and 25^th ^percentile of the individual average OD values; whiskers represent the maximum and minimum values. Differences between groups were tested using Mann-Whitney test. (B) ROC curve evaluating the threshold value of anti-SGS that separates non-infected individuals from asymptomatic *P. vivax *infection. Area under curve (AUC) calculated, together with the cut off value, which presents the higher likelihood ratio, and p values are plotted.

**Figure 3:**
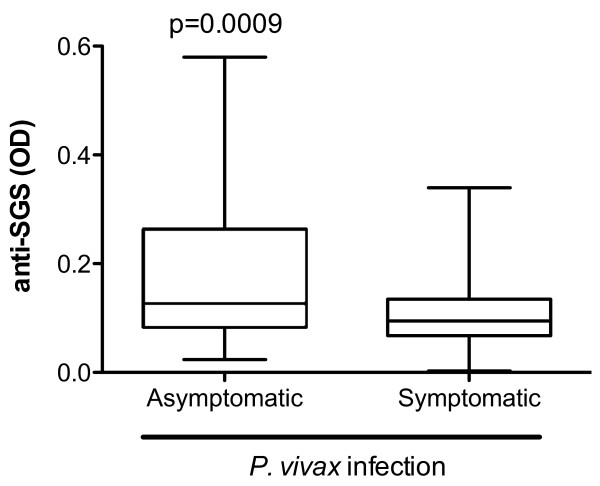
**Serum Anti-*An. darlingi *SGS levels in patients with different clinical spectrum of *P. vivax *infection**. Serums were collected from volunteers with asymptomatic *P. vivax *infection (n = 50) and from patients with symptomatic infection (n = 74). An ELISA test was performed to assess the IgG anti-*An. darlingi *SGS. Box plot graph, with lines of the boxes representing 75^th ^percentile, median and 25^th ^percentile of the individual average OD values; whiskers represent the maximum and minimum values. Differences between groups were tested using Mann-Whitney test; p value is plotted.

Diagnosis of symptomatic malaria cases is routinely performed in the endemic areas. A real challenge for diagnosis is to discriminate asymptomatic *Plasmodium*-infected individuals from those with no malaria infection. Despite presenting no clinical manifestations, asymptomatic *Plasmodium*-infected individuals are able to transmit *Plasmodium *to uninfected mosquitoes [22]. Thus, asymptomatic persons could serve as important reservoirs, and the possibility of identifying them could be useful for malaria control. Asymptomatic individuals presented higher anti-SGS antibody levels than non-infected individuals (Figure 2A, p < 0.0001). A ROC curve to discriminate these two clinical conditions showed that a cut-off value of 0.0935 OD, with a likelihood ratio of 3.03, indicated asymptomatic infection (Figure 2B; AUC: 0.798; p = 0.0001). Considering solely the *P. vivax *infected patients, asymptomatic individuals presented higher levels of anti-SGS than symptomatic ones (Figure 3, p = 0.0009). Evaluation of antibodies against *An. darlingi *saliva may serve as a marker of *P. vivax *asymptomatic infection in this Brazilian malaria endemic area.

**Figure 4:**
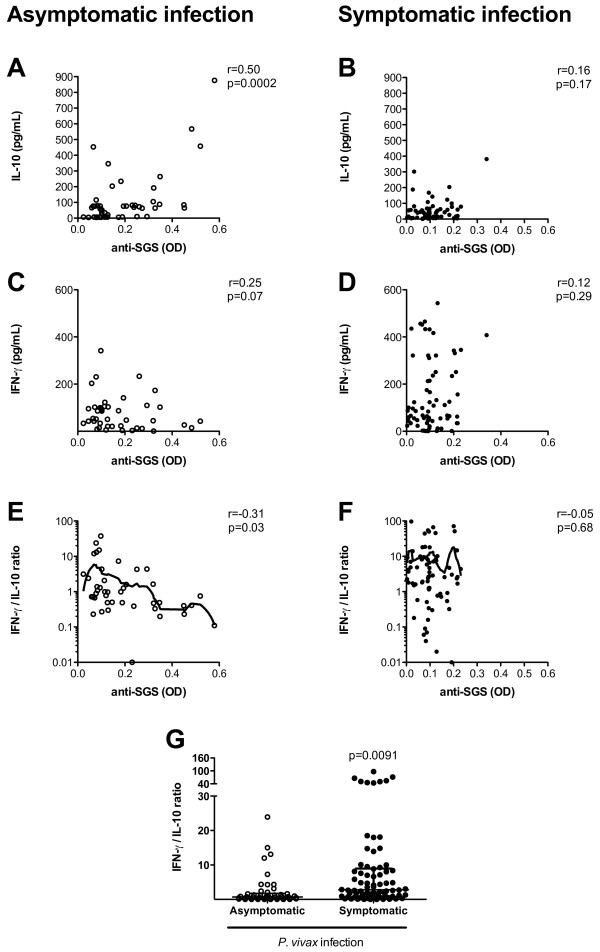
**Correlation between cytokine plasma levels and anti-saliva IgG titers**. (A) IL-10 vs. anti-SGS (OD), (B) IFN-γ, and (C) IFN-γ/IL-10 ratio vs. anti-SGS in patients with asymptomatic *P. vivax *infection. (D) IL-10 vs. anti-SGS (OD), (E) IFN-γ, and (F) IFN-γ/IL-10 ratio vs. anti-SGS in patients with symptomatic *P. vivax *infection. Fine Lowess curves are shown in (C) and (F) to evidence the trend of the data. Non-parametric Spearman test was used to verify statistical significance. (G) Comparison of IFN-γ/IL-10 ratio between volunteers with asymptomatic or symptomatic *P. vivax *infection. Mann-Whitney test was used to estimate the significance. P values, together with r values, are plotted in each graph.

In order to explore immunopathological patterns in *P. vivax *infection, the correlation between the anti-SGS antibody levels and the serological cytokine profile in asymptomatic and symptomatic malaria patients was evaluated. Volunteers with asymptomatic parasitaemia had a positive correlation between IL-10 and anti-SGS levels (Figure 4A. r = 0.50; p = 0.0002), but this finding was not seen in symptomatic individuals (Figure 4B. r = 0.16; p = 0.17). IFN-γ serum levels did not display significant correlation with anti-saliva antibodies in either asymptomatic (Figure 4C. r = 0.25; p = 0.07) or symptomatic (Figure 4D. r = 0.12; p = 0.29) patients. Moreover, a significant negative correlation between the IFN-γ/IL-10 ratio and anti-SGS levels in asymptomatic patients was noted (Figure 4E. r = -31; p = 0.03) but not in the symptomatic ones (Figure 4F. r = 0.05; p = 0.88). Thus, besides differing in anti-SGS antibody levels, asymptomatic and symptomatic *P. vivax*-infected individuals also differ in their cytokine balance. Cytokine profile may be implicated in minimizing *P. vivax *immunopathology, as individuals with asymptomatic infection presented lower IFN-γ/IL-10 ratio compared to symptomatic patients (Figure 4G; p < 0.0001). As previously described [3] and also presented in this work (Table 2), asymptomatic parasitaemia directly correlated to increased age and is more frequently observed in people residing for a long time in malaria endemic areas. In these regions, an extensive exposure to mosquito bites occurs over time. Malaria infection rates in these insects usually range from below 0.1% to 10% [23,24]. Consequently, each inhabitant is exposed to much more uninfected mosquitoes than infected ones. The recurrent exposure to mosquito bites or also to the *Plasmodium *may lead to a modification on the host immune response. It has been shown that repeated exposure to mosquito bites induces a Th1 profile in experimental models, leading to increased resistance to *Plasmodium *transmission [11]. This work shows that chronic exposure to *An. darlingi *bites relates to a reduction in the IFN-γ/IL-10 ratio not implying any causal relationship. On the other hand, as malaria clinical syndromes result from inadequate activation of pro-inflammatory cascades, oxidative stress and disturbs in immune regulation [25], this study hypothesizes that people residing in malaria endemic areas repeatedly exposed to uninfected mosquito bites over many years develop an efficient anti-saliva immune response, in which IL-10 may favor the production of specific antibodies. The neutralization of some vector salivary proteins may create microenvironment alterations in the site of mosquito bites that might ultimately affect the transmission of malaria. Another possibility is that mosquito bites, and also the continued exposure to *Plasmodium*, induce higher production of IL-10, which may reduce intense pro-inflammatory responses and the immunopathology of the infection. This study does not present experimental basis to indicate any direct effect of antibodies against *An. darlingi *salivary components on clinical status of *P. vivax *infected individuals. An important limitation regarding the use of anti-SGS levels as a marker for malaria infection is represented by the considerable variation of malaria transmission among different areas and also from season to season. This would make mandatory the establishment of appropriate anti-SGS cut-off level before using it as a marker for malaria. Nevertheless, once the cut-off levels are defined, the measurement of anti-SGS could serve as a very sensible indicator of this disease.

## Conclusion

Through the estimation of serum anti-*An. darlingi *saliva antibody levels, it is possible to infer the probable *P. vivax *infection status as marker of disease severity of an individual from the Amazon endemic area. Moreover, this study also suggests that the clinical immunity against *P. vivax *could be associated to a specific humoral response against the salivary components. As previously described to other vector-borne diseases, such as leishmaniasis, the detection of increased levels of anti-vector saliva could be pointed as an epidemiological marker of infection and also as a suitable indicator of clinical immunity in endemic regions.

## Competing interests

The authors declare that they have no competing interests.

## Authors' contributions

BBA designed the study, collected the serum samples, performed serology and cytokine experiments, the statistical analysis and drafted the manuscript. BCR, WPT, and LAM provided the salivary glands and helped in data analysis. ARF performed the SGS preparation and helped with the manuscript. LMAC participated in the design of the study, collected serum samples, examined the volunteers, and helped in data analysis. AB participated in the design of the study and helped in data analysis. MBN conceived of the study, participated in its design and coordination and helped in writing the manuscript. All authors have read and approved the final manuscript.

## Authors' information

BBA received a PhD fellowship and ARF a scientific initiation fellowship from the Brazilian National Research Council (CNPq). LAM, AB and MB-N are senior investigators from CNPq.

## References

[B1] Rogier C, Trape JF (1995). Study of premunition development in holo- and meso-endemic malaria areas in Dielmo and Ndiop (Senegal): preliminary results, 1990–1994. Med Trop (Mars).

[B2] Baird JK, Jones TR, Danudirgo EW, Annis BA, Bangs MJ, Basri H, Purnomo, Masbar S (1991). Age-dependent acquired protection against *Plasmodium falciparum *in people having two years exposure to hyperendemic malaria. Am J Trop Med Hyg.

[B3] Alves FP, Durlacher RR, Menezes MJ, Krieger H, Silva LH, Camargo EP (2002). High prevalence of asymptomatic *Plasmodium vivax *and *Plasmodium falciparum *infections in native Amazonian populations. Am J Trop Med Hyg.

[B4] Camargo LM, Noronha E, Salcedo JM, Dutra AP, Krieger H, Pereira da Silva LH, Camargo EP (1999). The epidemiology of malaria in Rondonia (Western Amazon region, Brazil): study of a riverine population. Acta Trop.

[B5] Ladeia-Andrade S, Ferreira MU, de Carvalho ME, Curado I, Coura JR (2009). Age-dependent acquisition of protective immunity to malaria in riverine populations of the Amazon Basin of Brazil. Am J Trop Med Hyg.

[B6] D'Ombrain MC, Robinson LJ, Stanisic DI, Taraika J, Bernard N, Michon P, Mueller I, Schofield L (2008). Association of early interferon-gamma production with immunity to clinical malaria: a longitudinal study among Papua New Guinean children. Clin Infect Dis.

[B7] Camargo EP, Alves F, Pereira da Silva LH (1999). Symptomless *Plasmodium vivax *infections in native Amazonians. Lancet.

[B8] Braga EM, Barros RM, Reis TA, Fontes CJ, Morais CG, Martins MS, Krettli AU (2002). Association of the IgG response to *Plasmodium falciparum *merozoite protein (C-terminal 19 kD) with clinical immunity to malaria in the Brazilian Amazon region. Am J Trop Med Hyg.

[B9] Bottius E, Guanzirolli A, Trape JF, Rogier C, Konate L, Druilhe P (1996). Malaria: even more chronic in nature than previously thought; evidence for subpatent parasitaemia detectable by the polymerase chain reaction. Trans R Soc Trop Med Hyg.

[B10] Peng Z, Simons FE (2004). Mosquito allergy: immune mechanisms and recombinant salivary allergens. Int Arch Allergy Immunol.

[B11] Donovan MJ, Messmore AS, Scrafford DA, Sacks DL, Kamhawi S, McDowell MA (2007). Uninfected mosquito bites confer protection against infection with malaria parasites. Infect Immun.

[B12] Orlandi-Pradines E, Almeras L, Denis de Senneville L, Barbe S, Remoue F, Villard C, Cornelie S, Penhoat K, Pascual A, Bourgouin C (2007). Antibody response against saliva antigens of *Anopheles gambiae *and *Aedes aegypti *in travellers in tropical Africa. Microbes Infect.

[B13] Remoue F, Cisse B, Ba F, Sokhna C, Herve JP, Boulanger D, Simondon F (2006). Evaluation of the antibody response to *Anopheles *salivary antigens as a potential marker of risk of malaria. Trans R Soc Trop Med Hyg.

[B14] Waitayakul A, Somsri S, Sattabongkot J, Looareesuwan S, Cui L, Udomsangpetch R (2006). Natural human humoral response to salivary gland proteins of *Anopheles *mosquitoes in Thailand. Acta Trop.

[B15] Pattanayak SK, Dickinson K, Corey C, Murray B, Sills E, Kramer R (2006). Deforestation, malaria, and poverty: a call for transdisciplinary research to support the design of cross-sectoral policies. Sustainability: Science, Pratice & Policy.

[B16] Rodrigues Ade F, Escobar AL, Souza-Santos R (2008). Spatial analysis and determination of malaria control areas in the State of Rondonia. Rev Soc Bras Med Trop.

[B17] da Silva J (2006). National System in Health Surveillance: situation report: Rondônia.

[B18] Cavasini MT, Ribeiro WL, Kawamoto F, Ferreira MU (2000). How prevalent is *Plasmodium malariae *in Rondonia, western Brazilian Amazon?. Rev Soc Bras Med Trop.

[B19] Snounou G (1996). Detection and identification of the four malaria parasite species infecting humans by PCR amplification. Methods Mol Biol.

[B20] Snounou G, Viriyakosol S, Zhu XP, Jarra W, Pinheiro L, do Rosario VE, Thaithong S, Brown KN (1993). High sensitivity of detection of human malaria parasites by the use of nested polymerase chain reaction. Mol Biochem Parasitol.

[B21] Deane LM (1986). Malaria vectors in Brazil. Mem Inst Oswaldo Cruz.

[B22] Alves FP, Gil LH, Marrelli MT, Ribolla PE, Camargo EP, Da Silva LH (2005). Asymptomatic carriers of *Plasmodium spp. *as infection source for malaria vector mosquitoes in the Brazilian Amazon. J Med Entomol.

[B23] Gil LH, Alves FP, Zieler H, Salcedo JM, Durlacher RR, Cunha RP, Tada MS, Camargo LM, Camargo EP, Pereira-da-Silva LH (2003). Seasonal malaria transmission and variation of anopheline density in two distinct endemic areas in Brazilian Amazonia. J Med Entomol.

[B24] Lines JD, Wilkes TJ, Lyimo EO (1991). Human malaria infectiousness measured by age-specific sporozoite rates in *Anopheles gambiae *in Tanzania. Parasitology.

[B25] Schofield L, Grau GE (2005). Immunological processes in malaria pathogenesis. Nat Rev Immunol.

